# COVID-19 Vaccine Acceptance among Low- and Lower-Middle-Income Countries: A Rapid Systematic Review and Meta-Analysis

**DOI:** 10.3390/vaccines10030427

**Published:** 2022-03-11

**Authors:** Muhammad Mainuddin Patwary, Md Ashraful Alam, Mondira Bardhan, Asma Safia Disha, Md. Zahidul Haque, Sharif Mutasim Billah, Md Pervez Kabir, Matthew H. E. M. Browning, Md. Mizanur Rahman, Ali Davod Parsa, Russell Kabir

**Affiliations:** 1 Environment and Sustainability Research Initiative, Khulna 9208, Bangladesh; mondirabardhan.22@gmail.com (M.B.); asmasafia.disha@ugent.be (A.S.D.); rtr.zahid@gmail.com (M.Z.H.); shakib8376@gmail.com (S.M.B.); mkabi086@uottawa.ca (M.P.K.); 2 Environmental Science Discipline, Life Science School, Khulna University, Khulna 9208, Bangladesh; 3 Department of Global Health Policy, Graduate School of Medicine, The University of Tokyo, Tokyo 113-0033, Japan; aalam@m.u-tokyo.ac.jp; 4 Tokyo Foundation for Policy Research, Tokyo 106-6234, Japan; 5 Department of Parks, Recreation and Tourism Management, Clemson University, Clemson, SC 29634, USA; mhb2@clemson.edu; 6 Hitotsubashi Institute for Advanced Study, Hitotsubashi University, 2-1 Naka Kunitachi, Tokyo 186-8601, Japan; mizanur.rahman@r.hit-u.ac.jp; 7 School of Allied Health, Faculty of Health, Education, Medicine and Social Care, Anglia Ruskin University, Chelmsford CM1 1SQ, UK; ali.parsa@aru.ac.uk (A.D.P.); russell.kabir@aru.ac.uk (R.K.)

**Keywords:** vaccine hesitancy, vaccine acceptance, COVID-19, low- and lower-middle income countries, meta-analysis, SARS-CoV-2, vaccine

## Abstract

Widespread vaccination against COVID-19 is critical for controlling the pandemic. Despite the development of safe and efficacious vaccinations, low-and lower-middle income countries (LMICs) continue to encounter barriers to care owing to inequitable access and vaccine apprehension. This study aimed to summarize the available data on COVID-19 vaccine acceptance rates and factors associated with acceptance in LMICs. A comprehensive search was performed in PubMed, Scopus, and Web of Science from inception through August 2021. Quality assessments of the included studies were carried out using the eight-item Joanna Briggs Institute Critical Appraisal tool for cross-sectional studies. We performed a meta-analysis to estimate pooled acceptance rates with 95% confidence intervals (CI). A total of 36 studies met the inclusion criteria and were included in the review. A total of 83,867 respondents from 33 countries were studied. Most of the studies were conducted in India (*n* = 9), Egypt (*n* = 6), Bangladesh (*n* = 4), or Nigeria (*n* = 4). The pooled-effect size of the COVID-19 vaccine acceptance rate was 58.5% (95% CI: 46.9, 69.7, *I*^2^ = 100%, 33 studies) and the pooled vaccine hesitancy rate was 38.2% (95% CI: 27.2–49.7, *I*^2^ = 100%, 32 studies). In country-specific sub-group analyses, India showed the highest rates of vaccine acceptancy (76.7%, 95% CI: 65.8–84.9%, *I*^2^ *=* 98%), while Egypt showed the lowest rates of vaccine acceptancy (42.6%, 95% CI: 16.6–73.5%, *I*^2^ *=* 98%). Being male and perceiving risk of COVID-19 infection were predictors for willingness to accept the vaccine. Increasing vaccine acceptance rates in the global south should be prioritized to advance global vaccination coverage.

## 1. Introduction

The highly infectious SARS-CoV-2 virus caused a worldwide outbreak, now known as coronavirus disease 2019 (COVID-19), and continues to present threats to nations across the globe. The manifestations of COVID-19 vary from person to person, from asymptomatic or moderate symptoms to a severe course of the illness [1]. The COVID-19 pandemic has taken a significant toll on the health of the globe in terms of incidence, mortality, mental health, and quality of life. As of 4 February 2022, the world crossed the threshold of 300 million COVID-19 cases and 5.7 million deaths [2]. Despite extensive research, there is currently no therapeutic for SARS-CoV-2 infection that has been proved to be consistently effective in controlled trials. The worldwide immunization against SARS-CoV-2 thus offers the possibility for a breakthrough in the battle against the severe effects of this emergent virus [1].

The 73rd World Health Assembly passed a resolution in May 2020 acknowledging the need for widespread vaccination as a nationwide public-health objective for preventing, controlling, and halting SARS-CoV-2 transmission [3]. Twenty-three vaccines have been authorized for emergency use in at least one country, 122 are in different clinical stages, and 194 are in pre-clinical developmental phases as of 15 November 2021 [4]. In most cases, the total effect of these vaccines is much higher than the threshold (50% efficacy) established by the U.S. Food and Drug Administration (FDA), including the BNT162b2 mRNA (95%), mRNA-1273 (94.1%), Sputnik V (91.6%) and the ChAdOx1 nCoV-19 (70.4%) vaccines.

Vaccination hesitancy and acceptance have emerged as prominent issues in the global fight against COVID-19 [5]. The Strategic Advisory Group of Experts (SAGE), a working group of the World Health Organization (WHO), has defined vaccine hesitancy as “the delay in acceptance or refusal of vaccines despite availability of vaccine service” [6]. According to a recent systematic review conducted in February 2021, the acceptability rate of COVID-19 vaccines ranged from 27.7% to 77.3 % [7]. Other studies have been conducted on predictors of receiving a vaccination. One study of 1268 respondents found 93% desired vaccination if the vaccine were 95% effective, while only 67% desired vaccination if it were 50% effective [8]. Even under the most effective vaccination scenarios, the majority of pregnant women (63%) and healthcare workers (34.35%) were hesitant to receive a vaccine in other studies [5,9]. Records of the H1N1 vaccination rollout among 18–75 year French residents showed declining rates of vaccine acceptance over time—from 90% in 2005 to 61% in 2010 [10]. During the 2018 measles epidemic in New York City, limited vaccine acceptance resulted in extensive and continuous disease transmission [5]. Consequently, the WHO has since listed vaccine hesitancy as one of the top 10 global health risks, even before the COVID-19 pandemic [11]. To ascertain the causes behind vaccine hesitancy, the WHO and United Nations (U.N.) Children’s Fund jointly performed a survey of 196 nations and found 74% of respondents had concerns about the risks and benefits of vaccines; further, these concerns were the most common sources of vaccine hesitancy [12]. Vaccination hesitation is widely recognized as a complicated phenomenon, with a variety of predictors beyond safety concerns. The Epidemiologic Triangle model can be applied to better understand vaccine hesitancy through three concepts that are related and interdependent: the environment, agent, and host. In this model, environmental factors may include social issues, public health policy, and the media. Agent factors may include perceptions of vaccine safety and efficacy, lack of trust in health systems, and perceived vulnerability of the disease. Host factors may include knowledge, education levels, previous experiences, and economic conditions [8,13,14].

Low- and lower-middle-income countries (LMICs) generally show higher willingness to accept vaccinations than higher-income countries [13,15,16]. For instance, Solís Arce et al. (2021) reported that 80% of LMIC samples were willing to accept vaccinations. Another study found an even greater proportion of willingness in South Asia (95%) [13]. In this case, the majority of LMIC respondents were willing to receive vaccinations because they believed vaccinations would protect them against COVID-19 [17]. However, there are studies reporting differing rates of vaccine hesitancy among other LMICs, such as India, Indonesia, Pakistan, and Burkina Faso [3,8,17]. A study among healthcare workers in Bangladesh reported that more than 50% of respondents were not willing to accept the COVID-19 vaccination. The major reasons for refusing vaccination were unknown side effects and perceived compromised quality as a result of the accelerated development of these vaccines [18]. A nationwide study in Pakistan found that more than half of participants expressed hesitancy toward COVID-19 vaccination [19]. Another study in Pakistan found that 38% of respondents expressed vaccine hesitancy and concerns about vaccine reliability and religious inhibitions [20]. A much higher proportion of participants (85%) disapproved of the COVID-19 vaccine in Cameroon and had doubts regarding its efficacy and safety, which influenced their attitudes toward the vaccine [21]. Similar findings were reported in Egypt, where 79% of respondents were vaccine hesitant [22].

As COVID-19 mortality rates in LMICs have been consistently lower than those in higher-income countries, LMIC residents might not acknowledge the risks of the disease and are, therefore, less willing to receive vaccines [15]. Poor knowledge, inadequate allocation of efficient vaccines, negative historical experiences involving foreign actors, cultural and religious beliefs, and mistrust in governments may explain vaccine hesitancy in LMICs [15,17]. However, we are unaware of comprehensive categorizations and confirmations of these factors and their effects. Earlier reviews have focused on summarizing global vaccine hesitancy rates [13], calculating global vaccine acceptance rates [23], pooling vaccine acceptance rates and their predictors [5], describing vaccine acceptance rates among LMICs in a qualitative fashion [24], scoping vaccine acceptance rates in higher-income countries [25], or scoping vaccine hesitancy rates and their predictors [26]. A systematic review with a meta-analysis of vaccine acceptance and hesitancy rates and their associated factors in LMICs has not yet been explored.

We conducted a rapid systematic review and meta-analysis aiming to estimate COVID-19 vaccine acceptance and hesitance rates among the people of LMICs. We also aimed to identify potential factors associated with vaccine acceptance in LMICs. As global vaccination efforts continue, this study could provide initial steps to facilitate the planning of ongoing vaccination programs and enhance vaccine uptake in developing countries.

## 2. Materials and Methods

We employed a rapid systematic review approach to synthesize the evidence using an expedited process [27]. The methodology was conducted according to the Preferred Reporting Items for Systematic Reviews and Meta-Analyses Statement (PRISMA) recommendations [28].

### 2.1. Search Strategy

A systematic search was carried out in Medline (via PubMed), Web of Science, and Scopus on 22 August 2021. We used medical subject headings (MeSH) and text words (tw) for the following search terms: (i) related to COVID-19—“COVID-19” OR “SARS-CoV-2” OR “coronavirus” OR “novel coronavirus” OR “nCoV” OR “2019-ncov” OR “SARS-CoV-2” OR “severe acute respiratory syndrome coronavirus 2”; (ii) related to vaccines—“vaccines” OR “vaccination” OR “COVID-19 Vaccines” OR “vaccina” OR “vaccine uptake” OR “SARS-CoV-2 vaccine”; (iii) related to acceptance or hesitancy—“Vaccine hesitancy” OR “vaccine hesitance” OR “Vaccine acceptance” OR “vaccine confidence” OR “Vaccine safety” OR “vaccination attitudes” OR “vaccine rejection” OR “vaccine willingness”; (iv) related to study design—“surveys and questionnaires” OR “survey” OR “poll” OR “surveys and questionnaires” OR “Cross-Sectional Studies”. The search strategies were developed for PubMed and revised for other databases. Other relevant articles were retrieved with forward and backward citation searches on the articles and reviews identified in the keyword searches via Google Scholar.

### 2.2. Study Selection

All records were imported to Rayyan (https://www.rayyan.ai/ accessed on 15 January 2022), an intelligent systematic review tool. After removing duplications, the complete contents of the relevant articles were reviewed for inclusion and exclusion criteria. The records were examined by five authors (MMP, MB, MZH, ASD, and SMB) based on study titles, abstracts, and full texts. Three independent reviewers (MMP, MB, and MZH) then assessed potentially eligible publications and resolved conflicts through discussion. Studies that matched the following criteria were eligible for inclusion: survey studies with no restrictions on the study population; descriptive and observational studies with a cross-sectional, experimental, or longitudinal design; at least one query on COVID-19 vaccine acceptance or hesitance; restricted to low-middle-income countries, as defined as a gross national income (GNI) per capita of USD 4095 or less in 2020 according to the World Bank; peer-reviewed studies published in English; published between January 2020 and August 2021. Articles that did not aim to evaluate COVID-19 vaccine acceptance/hesitancy were excluded. Literature reviews, systematic reviews, meta-analyses, unpublished data, books, conference papers, editorials, commentaries, letters to the editor, case reports were excluded. Studies reporting probable errors or results the reviewers were unable to extract correctly were also excluded, as were studies without full-text access.

### 2.3. Data Extraction

Data were extracted by four reviewers. The following pieces of information were retrieved: author-name; publication year; study country; study design; survey method and period; target population; sampling method; sample size; measurement scale of vaccine acceptance; statistical analysis; acceptance rate; unwillingness rate; hesitancy rate; factors associated with vaccine acceptance, hesitancy or refusal; reason for vaccine hesitancy or refusal; and summary of results. All extracted data are provided in Table 1. After independent data extraction, disparities were resolved by consensus.

### 2.4. Assessment of Study Quality

The Joanna Briggs Institute (JBI) critical appraisal tool was used to evaluate the quality of the included articles [56]. The checklist comprised of eight questions on the study design and data analysis (e.g., sample size, sample selection, valid and reliable measurements). The total score for each study was calculated by adding up the individual scores and putting them into groups based on previous studies [24,57,58], as displayed in Supplementary Table S1.

### 2.5. Data Analysis

The “Meta” and “Metasens” statistical packages in R version 4.2.1 were used for all analyses. To assess vaccine acceptance within subgroups, point estimates of effect size, odds ratios (ORs) and 95% confidence intervals (95% CI) were estimated. The pooled effects of vaccine acceptance and hesitance were calculated using random-effects models. Using meta-regression and subgroup analysis, the sources of heterogeneity were identified, and substantial heterogeneity was defined as an *I^2^* > 50% [59]. Begg’s test and Egger weighted-regression methods were adopted for calculating the presence and effect of publication bias.

## 3. Results

### 3.1. Search Results

**Figure 1:**
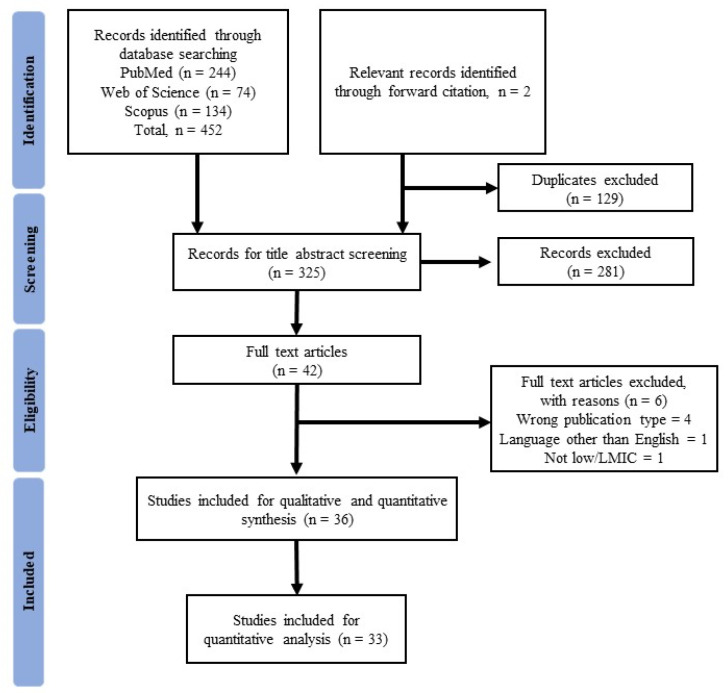
PRISMA flow diagram of the study selection process.

A total of 452 articles were identified in preliminary searches. After assessing eligibility based on the title and abstract or the full text, 36 articles were included in the final selection. Of these, four articles were found to contain results of multiple surveys. Lazarus et al. conducted a survey in 19 countries, but only three were from LMICs [46]. Bono et al. conducted an international study of nine LMICs [34]. Qunaibi et al. surveyed 23 Arab countries and territories and 122 other countries, of which eight were LMICs [52]. Lastly, Solís Arce et al. [15] performed an international study with 10 LMICs [15]. Of the total, 33 studies were included in the meta-analysis (Figure 1).

### 3.2. Characteristics of the Included Studies

Table 1 presents a synthesis of the included studies and factors of vaccine acceptance. Most studies had a cross-sectional design with data collected via telephone or online survey. Few studies recruited participants from existing databases, and some others used snowball sampling through social media or email, or convenience samples. All surveys were administered between March 2020 and April 2021. The total sample of included studies was 83,867, ranging from 187 participants [38] to 15,604 participants [15] in individual studies. Most of the studies were conducted in India (*n* = 9), Egypt (*n* = 6), Bangladesh (*n* = 4), or Nigeria (*n* = 4). The majority of the targeted samples were general populations, followed by healthcare workers and healthcare students. The most common factors for vaccine acceptance were older ages, gender, marital status, higher education levels, urban dweller, healthcare worker, chronic disease status, COVID-19 knowledge levels, perceived risk and benefits of vaccines, beliefs in vaccine safety and efficacy, previous vaccination history, and trust in healthcare systems.

### 3.3. Prevalence of Vaccine Acceptance and Hesitancy

**Figure 2:**
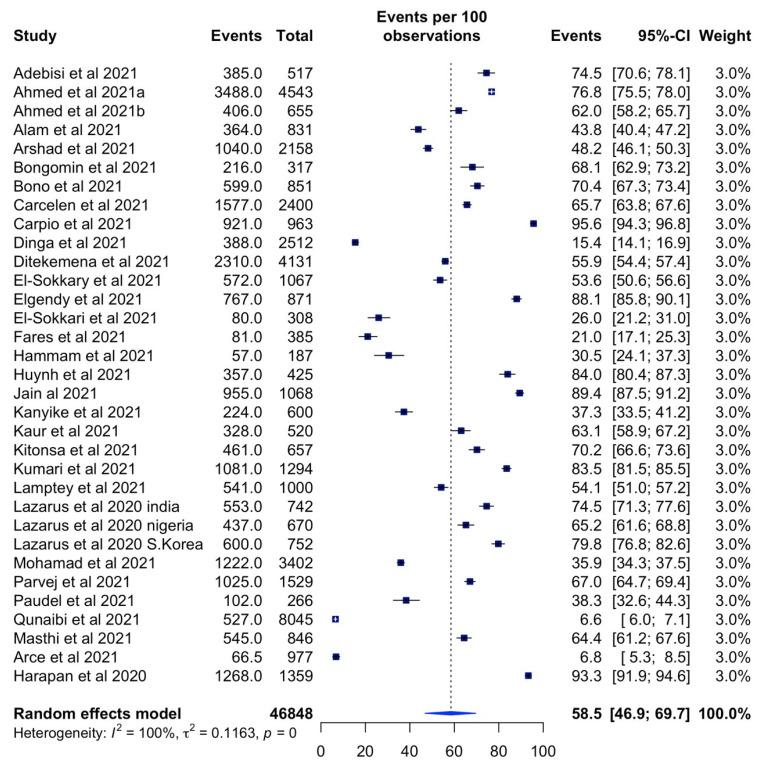
Forest plot of vaccine acceptance rates across LMICs.

The estimated COVID-19 vaccination acceptance rate across LMICs was 58.5% (95% CI: 46.90–69.70%, *I^2^* = 100%) (Figure 2). The highest rate was 95.6% (95% CI: 94.3–96.8%) in Kenya [34]. The study across eight LMICs by Qunaibi et al. [52] reported the lowest vaccination acceptance of 6.6% (95% CI: 6.0–7.1%).

**Figure 3:**
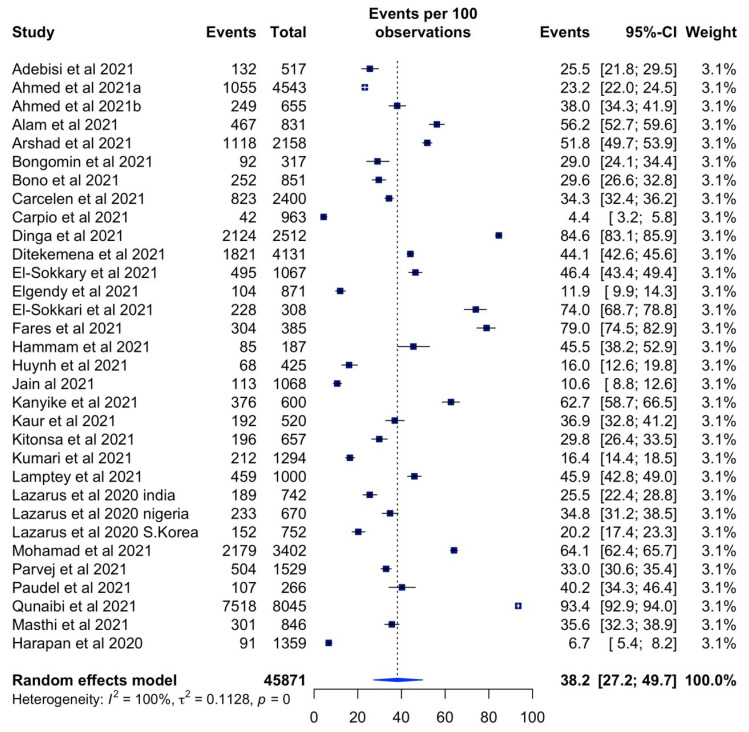
Forest plot of vaccine hesitancy rates across LMICs.

The total estimated COVID-19 vaccination hesitancy rate across LMICs was 38.2% (95% CI: 27.2–49.7%, *I^2^* = 100%) (Figure 3). The highest hesitancy rate was 84.6% (95% CI: 83.1–85.9%) in Cameroon [21] and the lowest rate was 4.4% (95% CI: 3.2–5.8%) in Kenya [34].

### 3.4. Sub-Group Analyses

**Figure 4:**
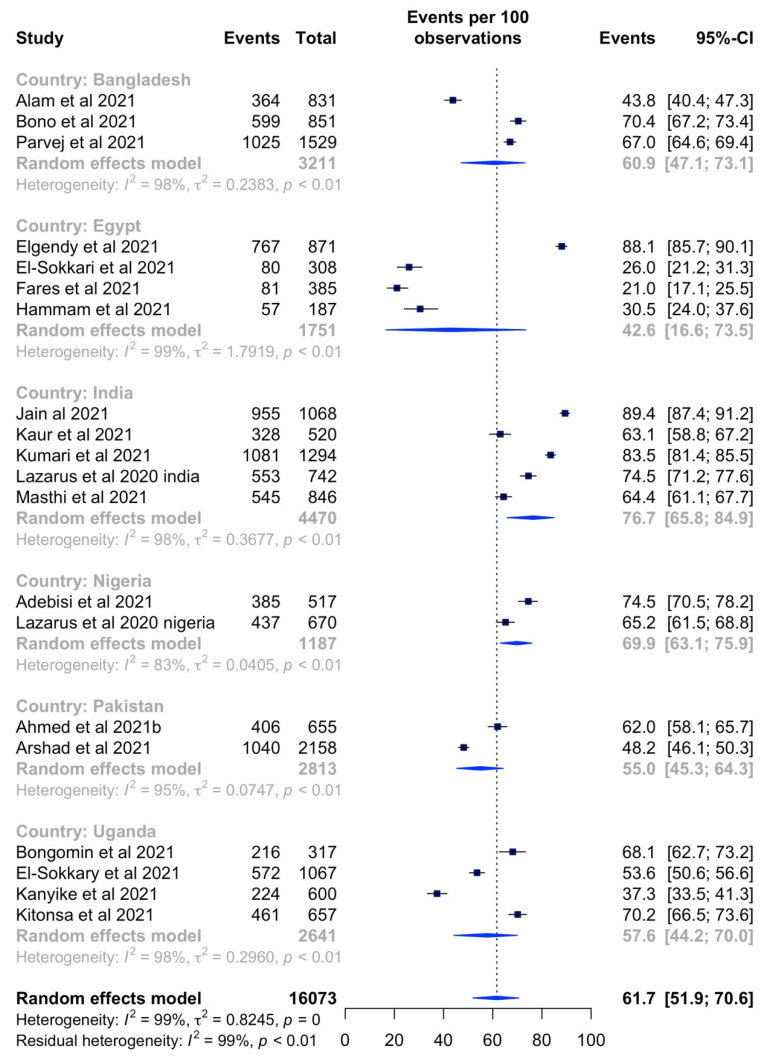
Forest plot of country-specific vaccine acceptance rates within LMICs.

In country-specific sub-group analyses, the pooled prevalence of the highest vaccine acceptance rates were observed in India (76.7%, 95% CI: 65.8–84.9%, *I^2^* = 98%) followed by Nigeria (69.9%, 95% CI: 63.1–75.9%, *I^2^* = 83%), Bangladesh (60.9%, 95% CI: 47.1–73.1%, *I^2^* = 98%), Uganda (57.6%, 95% CI: 44.2–70.0%, *I^2^* = 98%), Pakistan (55.0%, 95% CI: 45.3–64.3%, *I^2^* = 98%) and Egypt (42.6%, 95% CI: 16.6–73.5%, *I^2^* = 98%) (Figure 4).

**Figure 5:**
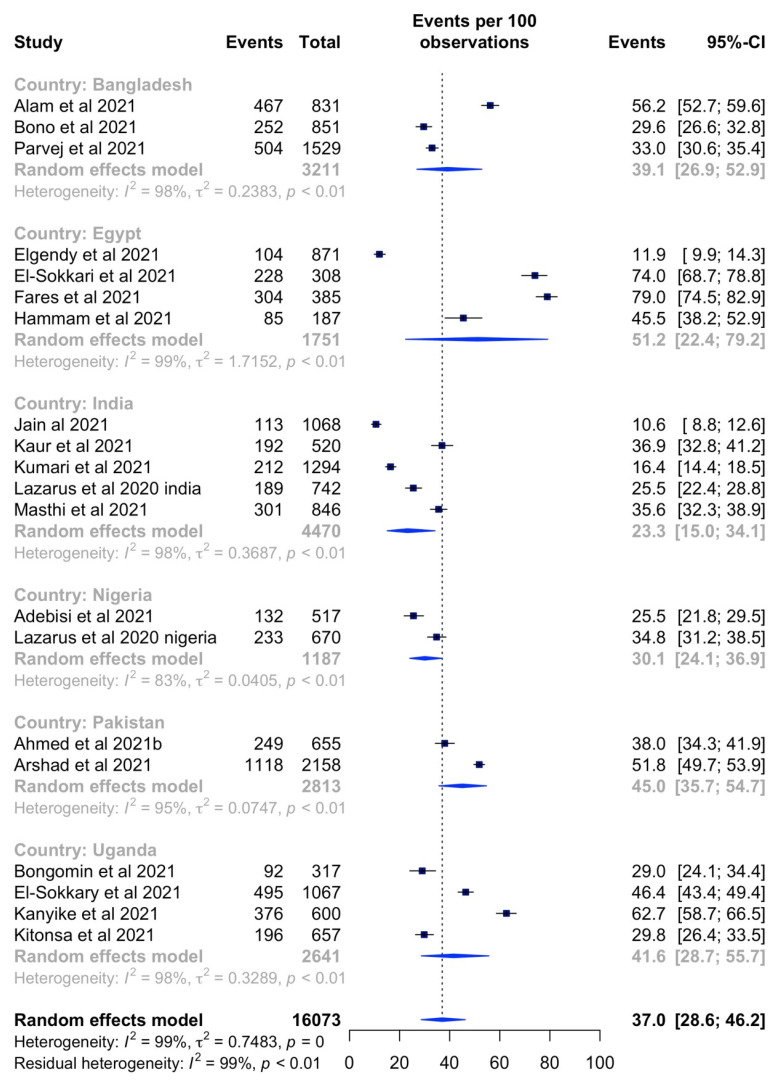
Forest plot of country-specific vaccine hesitancy rates within LMICs.

Additionally in country-specific sub-group analyses, the pooled prevalence of the highest COVID-19 vaccine hesitancy rates were observed in Egypt (51.2%, 95% CI: 22.4–79.2%, *I^2^* = 99%) followed by Pakistan (45.0%, 95% CI: 35.7–54.7%, *I^2^* = 95%), Uganda (41.6%, 95% CI: 28.7–55.7%, *I^2^* = 98%), Bangladesh (39.1%, 95% CI: 26.9–52.9%, *I^2^* = 98%), Nigeria (30.1%, 95% CI: 24.1–36.9%, *I^2^* = 83%), and India (23.3%, 95% CI: 15.0–34.1%, *I^2^* = 98%) (Figure 5).

**Figure 6:**
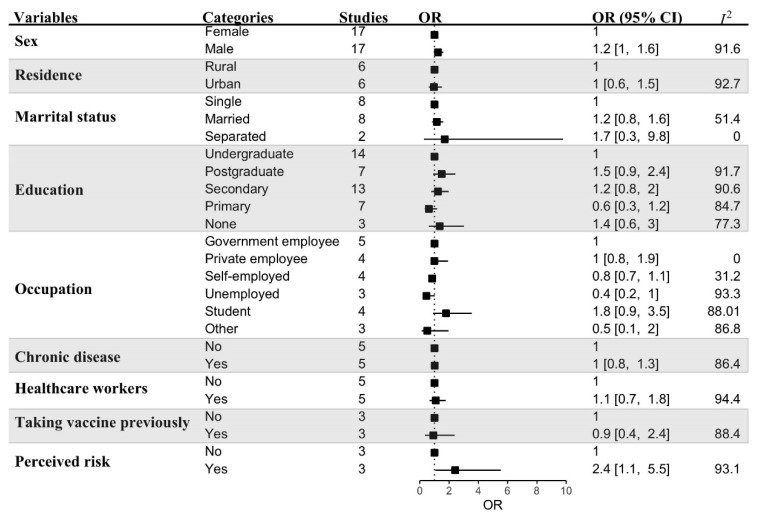
Meta-analysis of COVID-19 vaccination acceptance factors across LMICs.

Meta-estimates of COVID-19 vaccination acceptance rates and their factors are presented in Figure 6. Sex, residence, marital status, education, occupation, presence of chronic disease(s), healthcare worker status, previous vaccine history, and perceived risk of COVID-19 were checked as deterministic variables. Only being male (*n* = 17 studies, OR = 1.2, 95% CI: 1.0–1.6, *I^2^* = 91.6%) and perceived risk of COVID-19 infection (*n* = 3 studies, OR = 2.4, 95% CI = 1.1–5.5, *I^2^* = 93.1%) had high pooled odds ratios that were significantly associated with vaccination acceptance.

### 3.5. Risk of Bias

The JBI tool indicated no studies should be eliminated because of low methodological quality. However, many studies were identified as being reliant on inadequate recruitment methods such as convenience and snowball sampling via social media, which may not have produced representative samples. Regardless, each of the 36 studies was categorized into the high-quality category for observational studies (Supplementary Table S1).

We observed the presence of some publication bias. The results of the Egger’s test showed that studies of vaccine acceptance (Egger’s *p*-value = 0.02) and hesitancy (Egger’s *p*-value = 0.007) were vulnerable to publication bias (Supplementary Table S2, Figures S1 and S2).

## 4. Discussion

The COVID-19 pandemic has presented an unprecedented threat to public health [60]. To date, it has spread across more than 200 countries and has yet to be effectively controlled. Governments have adopted several strategies to control the spread of the infection; however, none have been entirely successful in stopping the epidemic [61]. Fortunately, several effective and safe COVID-19 vaccines have been developed. The current situation of the pandemic is now focused on the global need to slow the spread through vaccination rollouts. While vaccine acceptance is crucial to herd immunity, vaccine hesitancy is a major barrier to achieving this target, particularly in low-middle-income countries (LMICs) [52]. To the best of our knowledge, there is no systematic review and meta-analysis on estimating vaccine acceptance rates in LMICs. Therefore, the current study determined this rate and its factors LMICs using all available data as of August 2021.

We identified 36 studies of 83,867 participants from 33 low-middle-income countries. Pooled estimates showed that more than half of these participants were willing to accept the COVID-19 vaccine. India and Egypt reported the highest and lowest vaccine acceptance rates, respectively. Correspondingly, Egypt and India reported the highest and lowest vaccine hesitancy rates, respectively.

Our findings are different from other review articles on COVID-19 vaccine acceptance, which may be attributable to the extended timing of the published articles in the current review. The observed vaccine acceptance rate of 59% is much lower than global estimates of 66% by Nehal et al. [23] and 73% by Wang et al. [5]. However, these other reviews included studies only through April 2021. Other reviews have shown vaccine acceptance rates have varied over the course of the pandemic [23]. For example, a global review found acceptance rates increased from 57% in April 2020 to 75% in June 2020 in the U.S. [13]. Neumann-Böhme et al. [62] conducted a multi-country study in Europe and variability of vaccine acceptance was observed across countries during the time periods studied. Meanwhile, our study observed variation across studies due to high heterogeneity (*I^2^* = 100%) that was similar to rates in Nehal et al. [23] (99.4%) and Wang et al. [5] (98.8%).

Although vaccine distribution is low in LMICs, acceptance rates are high compared to high-income countries. An analysis recently reported in *Nature Medicine* found that 80% of participants from 10 LMICs were willing to accept the COVID-19 vaccine, while only 65% of participants in the U.S. were willing to accept the vaccine; in the upper-middle income country of Russia, only 30% of respondents were willing to accept the vaccine [15]. Vaccine acceptance rates among LMICs are less impacted by vaccine access, costs, and vaccine awareness than in high-income countries, where people tend to show hesitancy due to concerns about safety caused by the rapid pace of vaccine development [63]. Instead, the moderate levels of vaccine hesitancy in the current study might be explained by the low severity of COVID-19 cases in LMICs [64], negative perceptions of healthcare quality [65], exposure to widespread misinformation in social media [66], and low trust in governmental agencies. A prior study among healthcare workers in Bangladesh reported that unknown side effects of vaccines and the compromised quality of vaccines as a result of their accelerated development were reasons for vaccine hesitancy [18]. Another study in Pakistan found concerns regarding vaccine reliability and religious inhibition influenced vaccine acceptance rates [20]. Concerns about vaccine safety and effectiveness were also reported as major reasons for hesitancy in Cameroon and Egypt due to the rapid development of the vaccine [21,22].

The observed higher vaccine acceptance rates in India may be due to the high rates of COVID-19 mortality in that country, totaling more than 30 million cases with 0.4 million deaths [2]. India has also recorded an increasing rate of transmission with a basic reproduction number (R0) estimate of 2.69 [67]. This could be the reason why people in India reported high levels of fear about contracting COVID-19 infection and positive attitudes about vaccine efficacy and safety [15].

Low acceptance and high hesitancy were observed in African countries (e.g., Egypt, Uganda). One explanation is that the participating African countries observed lower rates of COVID-19 mortality. There is also a widespread belief that African countries are less susceptible to COVID-19, which raised doubt as to the need for additional investments in vaccinations in these countries [32]. People in Africa have historically had higher vaccine hesitancy rates, which could play a role in the observed acceptance rates for COVID-19 vaccines [68]. One prominent example is the Nigerian boycott of the polio vaccine during the early 2000s. Religious and political leaders feared that the vaccine could be deliberately contaminated with anti-fertility agents and HIV virus [69]. Also, widespread misconceptions and misinformation about COVID-19 have been across Africa. Many African communities also have poor health-seeking behaviors due to spiritual considerations, which could reduce vaccine uptake [70].

Our finding that males and perceived risk of COVID-19 were significant predictors of vaccine acceptance in LMICs is supported by other research. An earlier global systematic review and meta-analysis also found that men were more likely to accept the COVID-19 vaccine than women. One explanation could be the involvement of men in riskier behavior than women [71]. Alternatively, women may have lower levels of social support and be less responsive to healthcare communities and preventive measures than men [72]. Females may also be vaccine-hesitant due to lower COVID-19-related risk perceptions [73]. Regarding our risk perception findings, similar results were observed in another recent systematic review and meta-analysis [74]. Studies in Asia have also shown that positive attitudes toward vaccination were correlated with COVID-19 risk [75,76]. A study of Congo Healthcare workers reported high perceived risk of COVID-19 was also associated with greater intentions to receive a vaccination [68]. Such findings could be explained by the Health Belief Model (HBM), which suggests individuals who fear COVID-19 are more willing to get vaccinated due to the perceived benefits [77]. Participants who have tested negative for COVID-19 have also shown a greater likelihood of vaccine acceptance, and COVID-19 testing is often mandatory when people have had close contact with suspected or confirmed cases or family members have presented flu-like symptoms [78]. Thus, despite testing negative, awareness of the virus may increase as a result of testing experiences and ultimately increase vaccine acceptance [32].

### 4.1. Implications and Future Research Needs

Limited healthcare capacity and over-population make LMICs highly susceptible to COVID-19. It is a high priority for every government to ensure high vaccination rates to mitigate the transmission of the virus. Understanding public attitudes and views on vaccination are critical for meeting immunization requirements. The current systematic review and meta-analysis could provide guidance on steps forward, given our reported within and across-country estimates on vaccine acceptance and hesitancy. We suggest that country-specific interventions be taken to increase the acceptance rates in LMICs. In this sense, the government of each country should establish public faith in vaccines at the national level. At the same time, governments should be aware of anti-vaccination movements among people in LMICs resulting from misconception and misinformation in social media or other factors (i.e., spiritual ones) since these could limit vaccine acceptance [79]. Understanding the factors that may influence vaccination intentions (i.e., being male, perceived risk of COVID-19) may also allow greater effectiveness of vaccination programs.

The current review prompts future research needs on COVID-19 vaccine acceptance in LMICs. The included articles and sampled populations were restricted to a limited period; however, public attitudes toward vaccine hesitancy likely vary with time. Considering the ongoing waves of outbreaks in different countries, future research should focus on longitudinal changes in COVID-19 vaccine hesitancy in LMICs. In this case, our study provides initial guidance to understanding patterns in vaccination acceptance over time. Most of the studied populations in the included studies were from the general population. Future studies should also focus on estimating vaccine acceptance rates and determining underlying hesitancy factors among other groups, such as healthcare workers, pregnant women, children, and patients with chronic disease.

### 4.2. Strengths and Limitations

This study has several strengths. To the best of our knowledge, it is the first comprehensive meta-analysis on vaccine acceptance in LMICs. The included articles were also high-quality observational studies. We searched several well-known databases and the reference lists of the included studies and estimated vaccine acceptance rates from 33 studies of LMICs, which are under-represented in the literature on COVID-19 vaccinations. Our target populations were adults, regardless of any profession, and thus captured a wide range of populations.

However, our study also had some limitations. We only considered peer-reviewed articles published up to August 2021 and did not consider preprints or reports that were yet to be peer-reviewed. Given the surge in the number of COVID-19 publications, including grey literature or preprints could have resulted in a larger sample and different conclusions. Our data analysis covered studies across 2020 and 2021 despite public attitudes toward vaccination varying across this time period. For example, a study on older adults’ vaccine hesitancy observed rates of 14% during early 2020 and 24.0% during late 2020 [80]. Studies included in the current review were also mostly cross-sectional and used online surveys due to COVID-19 restrictions. Such findings from online surveys are subject to self-selection bias [80]. Finally, we could not consider all possible determinants of vaccine acceptance due to data limitations.

## 5. Conclusions

In a review of 33 articles, we found over 50% of LMIC residents were willing to accept the COVID-19 vaccine. India and Egypt reported the highest and lowest vaccine acceptance rates, respectively, while Egypt reported the highest vaccine hesitancy rate. Being male and perceiving risk of COVID-19 infection predicted willingness to accept the COVID-19 vaccine. Policymakers at national and sub-national levels should recognize gender and perceived risks toward COVID-19 as vaccine determinants. Vaccine hesitancy could be addressed by community leaders, community mobilization efforts, health care professional training, non-monetary incentives, and mass media campaigns to enhance knowledge and awareness about vaccinations and immunization. Prioritizing vaccine distribution in LMICs could yield significant gains in global vaccination coverage.

## Figures and Tables

**Table 1:** Characteristics of included studies.

SL	Author	Country	Study Design	Survey Period	Target Population	Sample Size, *n*	Vaccine Acceptance (%)	Factors Associated with Vaccine Acceptance
1	Adebisi et al. [29]	Nigeria	Cross-sectional	August 2020	General population	517	74.47	Age, geopolitical location, education level.
2	Ahmed et al. [30]	Somalia	Cross-sectional	26 December 2020–28 January 2021	General population	4543	76.78	Female, living in Galmudug, Hirshabelle and Southwest, student, worker in the healthcare sector, adherence score, presence of flu symptoms.
3	Ahmed et al. [20]	Pakistan	Cross-sectional	April 2021	General population	655	61.98	Older age, sometimes/not following Anti-COVID-19 SOPs, high chance of being infected, vaccination having the potential of preventing COVID-19 spread, observing the effects of the vaccine on others, knowing more about the vaccine, belief that a Muslim’s trust in God was sufficient to protect one from infection, the vaccine was prepared in a hurry without sufficient testing and could harm those with low immunity, seeing everyone else getting vaccinated, pressure from friends and family.
4	Akiful Haque et al. [17]	Bangladesh	Cross-sectional	17 January–2 February 2021	General population	7357	65.05	Graduates or above, age ≥ 50 years, students, monthly income ≥ 41,000 BDT, rural resident, respondents from Khulna division, family members diagnosed with COVID-19, presence of chronic disease, vaccinated in the last few years.
5	Alam et al. [18]	Bangladesh	Cross-sectional	3–25 January 2021	Healthcare professionals	831	43.80	Female, 18–34 age group, work in public/government institutes, nurses, not having received the flu vaccine in the previous year.
6	Arshad et al. [19]	Pakistan	Cross-sectional	January 2021	General population	2158	48.19	Gender, age, marital status, education level, occupation, profession, monthly income, residential area, myths, conspiracy beliefs.
7	Bongomin et al. [31]	Uganda	Cross-sectional	29 March–14 April 2021	General population	317	68.14	Female, patients who agreed or strongly agreed that they had some immunity against COVID-19, patients who had a history of vaccine hesitancy for their children.
8	Bono et al. [32]	Bangladesh	Cross-sectional	10 December 2020–9 February 2021	General population	230	89.57	COVID-19 knowledge, worry/fear regarding COVID-19, higher income, younger age, testing negative for COVID-19.
		DR Congo				219	59.36	
		Benin				159	48.43	
		Uganda				107	88.79	
		Malawi				81	61.73	
		Mali				55	74.55	
9	Bono et al. [32]	The Democratic Republic of Congo	Cross-sectional	24 August–8 September 2020	General population	4131	55.92	Middle or high-income, being tested for COVID-19, COVID-19 community vaccine acceptance, acknowledging the existence of COVID-19, healthcare worker.
10	Carcelen et al. [33]	Zambia	Cross-sectional	23–29 November 2020	Caregivers	2400	65.71	Belief in the COVID-19 vaccine safety and efficacy.
11	Carpio et al. [34]	Kenya	Cross-sectional	7–15 April 2020	General population	963	95.64	Vaccine duration of protection and efficacy, perceived probability of being hospitalized, age, gender, education, location, region of residence, household income.
12	Dinga et al. [21]	Cameroon	Cross-sectional	May–August 2020	General population	2512	15.45	NR *
13	Echoru et al. [35]	Western Uganda	Cross-sectional	July–September 2020	General population	1067	53.61	Younger, male, tertiary level of students, Muslims, married, on-salary earners, rural dwellers.
14	Elgendy and Abdelrahim [36]	Egypt	Cross-sectional	April–May 2021	General population	871	88.06	NR
15	El-Sokkary et al. [37]	Egypt	Cross-sectional	25–31 January 2021	Healthcare professionals	308	25.97	Income, years of experience.
16	Fares et al. [22]	Egypt	Observational	December 2020–January 2021	Healthcare professionals	385	21.04	Male, interacting directly with COVID-19 patients, taking non-compulsory vaccines, recommending COVID-19 vaccination to others, receiving advice from hospitals to get the vaccine, trust in vaccine producers, pharmaceutical companies, and authorities.
17	Hammam et al. [38]	Egypt	Cross-sectional	April 2021	Healthcare professionals	187	30.48	NR
18	Harapan et al. [8]	Indonesia	Cross-sectional	25 March–6 April 2020	General population	1359	93.30	Female, middle-aged, retired, married, healthcare worker, moderate perceived risk of COVID-19 infection.
19	Huynh et al. [39]	Vietnam	Cross-sectional	December 2020–January 2021	General population	425	84.00	Knowledge of COVID-19, cues to action toward the vaccine.
20	Jain et al. [40]	India	Cross-sectional	2 February–7 March 2021	Healthcare students	1068	89.42	NR
21	Kanyike et al. [41]	Uganda	Cross-sectional	15–21 March 2021	Healthcare students	600	37.33	Male, being single, very high or moderate perceived risk of contracting COVID-19, receiving any vaccine in the past five years, COVID-19 vaccine hesitancy,
22	Kaur et al. [42]	India	Cross-sectional	January 2021	Healthcare professionals	520	63.08	Dental professional, involved in COVID-19 duties, preference for natural immunity over the vaccine, belief in COVID-19 vaccine safety, interest in vaccine information, belief that vaccine should be compulsory.
23	Kitonsa et al. [43]	Uganda	Cross-sectional	September–November 2020	Healthcare professionals	657	70.17	NR
24	Kumari et al. [44]	India	Cross-sectional	13–25 March 2021	General population	1294	83.54	Older, belief that the vaccine is harmless, belief that vaccine benefits outweigh the risks, belief that getting vaccinated is a societal responsibility, belief that sufficient data about the vaccine is available, belief that the vaccine will eradicate COVID-19, role model getting vaccinated, many other people getting vaccinated, higher socioeconomic status, developed place of residence.
25	Lamptey et al. [45]	Ghana	Cross-sectional	14 October–12 December 2020	General population	1000	54.10	Being married, government worker, high-risk perceptions.
26	Lazarus et al. [46]	India	Cross-sectional	16–20 June 2020	General population	742	74.53	Male, older, higher education.
		Nigeria				670	65.22	
		South Korea				752	79.79	
27	Lazarus et al. [47]	South Korea	Cross-sectional	16–20 June 2020	General population	619 to 773	79.79	NR
		India					74.53	
		Nigeria					65.22	
28	Mohamad et al. [48]	Syria	Cross-sectional	23 December 2020–5 January 2021	General population	3402	35.82	Female, younger, urban resident, not married, no kids, not a healthcare worker, not a smoker, no fear of COVID-19, perceived severity of COVID-19, belief in the natural origin of the virus, knowledge on vaccine hesitancy.
29	Panda et al. [49]	India	Cross-sectional	February 2021	General population	359	8.08	NR
30	Parvej et al. [50]	Bangladesh	Cross-sectional	17–26 April 2021	General population	1529	67.04	Muslim, highly educated, living in urban areas, believing vaccines protect against infectious diseases and vaccines, having no health-related risks.
31	Paudel et al. [51]	Nepal	Cross-sectional	27 January–3 February 2021	Healthcare professionals	266	38.35	NR
32	Qunaibi et al. [52]	Algeria	Cross-sectional	14–29 January 2021	General population	2706	3.62	Receiving the influenza vaccine regularly, health care worker, resident in country with higher rates of COVID-19 infections.
		Egypt				5339	8.04	
		Mauritania				99	8.08	
		Morocco				3775	7.89	
		Sudan				313	15.34	
		Syria				1232	10.71	
		Tunisia				665	6.47	
		Yemen				226	9.29	
33	Ramesh Masthi and Sowmyashree [53]	India	Cross-sectional	January 2021	General population	846	64.42	NR
34	Saied et al. [54]	Egypt	Cross-sectional	8–15 January 2021	Healthcare students	2133	34.79	Pharmacy student, higher academic year or graduate, average to very good self-perception of health status, good self-rated COVID-19 knowledge level, presence of confirmed COVID-19 infection in a close social network.
35	Skjefte et al. [55]	India	Cross-sectional	28 October–18 November 2020	Pregnant women, mothers of young children	1639	Pregnant women (52) Non-pregnant woman (73.4)	NR
		Philippines				1034	NR	
36	Solis Arce et al. [15]	Burkina Faso	Cross-sectional	15 October–4 December 2020	General population	977	66.53	Protection for self, family, and community, recommendation from health workers and government.
		India		17 June 2020–18 January 2021	General population	1680	84.29	
		Mozambique		30 October–30 November 2020	General population	862	89.10	
		Nepal		1–11 December 2020	General population	1389	96.62	
		Nigeria		18 November–18 December 2020	General population	1868	76.18	
		Pakistan 1		24 July–9 September 2020	General population	1633	76.12	
		Pakistan 2		2 September–13 October 2020	General population	1492	66.49	
		Rwanda		22 October–15 November 2020	General population	1355	84.87	
		Sierra Leone 1		2–19 October 2020	General population	1070	78.04	
		Sierra Leone 2		7 October 2020–20 January 2021	General population	2110	87.91	
		Uganda 1		21 September–12 December 2020	General population	3362	85.81	
		Uganda 2		23 November–12 December 2020	General population	1366	76.50	

* NR, Not Reported.

## Data Availability

Data generated in this study is available by contacting the first author, M.M.P. if requested reasonably.

## Supplementary Materials

The following supporting information can be downloaded at: https://www.mdpi.com/article/10.3390/vaccines10030427/s1, Figure S1: Funnel plot of vaccine acceptance. Figure S2: Funnel plot of vaccine hesitance.; Table S1: Risk of bias for included studies in the systematic review. Table S2: Tests for publication bias.

## References

[1] Grochowska M., Ratajczak A., Zdunek G., Adamiec A., Waszkiewicz P., Feleszko W. (2021). A comparison of the level of acceptance and hesitancy towards the influenza vaccine and the forthcoming COVID-19 vaccine in the medical community. Vaccines.

[2] Worldometer COVID Live—Coronavirus Statistics—Worldometer. https://www.worldometers.info/coronavirus/.

[3] Machingaidze S., Wiysonge C.S. (2021). Understanding COVID-19 vaccine hesitancy. Nat. Med..

[4] Eroglu B., Nuwarda R.F., Ramzan I., Kayser V. (2022). A Narrative Review of COVID-19 Vaccines. Vaccines.

[5] Wang Q., Yang L., Jin H., Lin L. (2021). Vaccination against COVID-19: A systematic review and meta-analysis of acceptability and its predictors. Prev. Med..

[6] Dubé E., Bettinger J., Fisher W., Naus M., Mahmud S., Hilderman T. (2016). Improving Vaccination Rates: Vaccine acceptance, hesitancy and refusal in Canada: Challenges and potential approaches. Canada Commun. Dis. Rep..

[7] Li M., Luo Y., Watson R., Zheng Y., Ren J., Tang J., Chen Y. (2021). Healthcare workers’ (HCWs) attitudes and related factors towards COVID-19 vaccination: A rapid systematic review. Postgrad. Med. J..

[8] Harapan H., Wagner A.L., Yufika A., Winardi W., Anwar S., Gan A.K., Setiawan A.M., Rajamoorthy Y., Sofyan H., Mudatsir M. (2020). Acceptance of a COVID-19 Vaccine in Southeast Asia: A Cross-Sectional Study in Indonesia. Front. Public Health.

[9] Ayhan S.G., Oluklu D., Atalay A., Beser D.M., Tanacan A., Tekin O.M., Sahin D. (2021). COVID-19 vaccine acceptance in pregnant women. Int. J. Gynecol. Obstet..

[10] Charron J., Gautier A., Jestin C. (2020). Influence of information sources on vaccine hesitancy and practices. Médecine Mal. Infect..

[11] Feemster K.A. (2020). Building vaccine acceptance through communication and advocacy. Hum. Vaccin. Immunother..

[12] MacDonald N.E., Butler R., Dubé E. (2017). Addressing barriers to vaccine acceptance: An overview. Hum. Vaccin. Immunother..

[13] Sallam M. (2021). COVID-19 vaccine hesitancy worldwide: A concise systematic review of vaccine acceptance rates. Vaccines.

[14] Dubé È., Farrands A., Lemaitre T., Boulianne N., Sauvageau C., Boucher F.D., Tapiero B., Quach C., Ouakki M., Gosselin V. (2019). Overview of knowledge, attitudes, beliefs, vaccine hesitancy and vaccine acceptance among mothers of infants in Quebec, Canada. Hum. Vaccines Immunother..

[15] Solís Arce J.S., Warren S.S., Meriggi N.F., Scacco A., McMurry N., Voors M., Syunyaev G., Malik A.A., Aboutajdine S., Adeojo O. (2021). COVID-19 vaccine acceptance and hesitancy in low- and middle-income countries. Nat. Med..

[16] Patwary M.M., Bardhan M., Disha A.S., Hasan M., Haque M.Z., Sultana R., Hossain M.R., Browning M.H.E.M., Alam M.A., Sallam M. (2021). Determinants of COVID-19 Vaccine Acceptance among the Adult Population of Bangladesh Using the Health Belief Model and the Theory of Planned Behavior Model. Vaccines.

[17] Akiful Haque M.M., Rahman M.L., Hossian M., Matin K.F., Nabi M.H., Saha S., Hasan M., Manna R.M., Barsha S.Y., Hasan S.M.R. (2021). Acceptance of COVID-19 vaccine and its determinants: Evidence from a large sample study in Bangladesh. Heliyon.

[18] Alam A.B.M.M., Majumder M.A.A., Haque M., Ashraf F., Khondoker M.U., Mashreky S.R., Wahab A., Siddiqui T.H., Uddin A., Joarder T. (2021). Disproportionate COVID-19 vaccine acceptance rate among healthcare professionals on the eve of nationwide vaccine distribution in Bangladesh. Expert Rev. Vaccines.

[19] Arshad M.S., Hussain I., Mahmood T., Hayat K., Majeed A., Imran I., Saeed H., Iqbal M.O., Uzair M., Rehman A.U. (2021). A National Survey to Assess the COVID-19 Vaccine-Related Conspiracy Beliefs, Acceptability, Preference, and Willingness to Pay among the General Population of Pakistan. Vaccines.

[20] Ahmed T.F., Ahmed A., Ahmed S., Ahmed H.U. (2021). Understanding COVID-19 vaccine acceptance in Pakistan: An echo of previous immunizations or prospect of change?. Expert Rev. Vaccines.

[21] Dinga J.N., Sinda L.K., Titanji V.P.K. (2021). Assessment of vaccine hesitancy to a COVID-19 vaccine in Cameroonian adults and its global implication. Vaccines.

[22] Fares S., Elmnyer M.M., Mohamed S.S., Elsayed R. (2021). COVID-19 Vaccination Perception and Attitude among Healthcare Workers in Egypt. J. Prim. Care Community Health.

[23] Nehal K.R., Steendam L.M., Ponce M.C., van der Hoeven M., Smit G.S.A. (2021). Worldwide vaccination willingness for COVID-19: A systematic review and meta-analysis. Vaccines.

[24] Moola S., Gudi N., Nambiar D., Dumka N., Ahmed T., Sonawane I.R., Kotwal A. (2021). A rapid review of evidence on the determinants of and strategies for COVID-19 vaccine acceptance in low- and middle-income countries. J. Glob. Health.

[25] Aw J., Seng J.J.B., Seah S.S.Y., Low L.L. (2021). COVID-19 vaccine hesitancy—A scoping review of literature in high-income countries. Vaccines.

[26] Biswas M.R., Alzubaidi M.S., Shah U., Abd-Alrazaq A.A., Shah Z. (2021). A scoping review to find out worldwide COVID-19 vaccine hesitancy and its underlying determinants. Vaccines.

[27] Haby M.M., Chapman E., Clark R., Barreto J., Reveiz L., Lavis J.N. (2016). What are the best methodologies for rapid reviews of the research evidence for evidence-informed decision making in health policy and practice: A rapid review. Health Res. Policy Syst..

[28] Page M.J., McKenzie J.E., Bossuyt P.M., Boutron I., Hoffmann T.C., Mulrow C.D., Shamseer L., Tetzlaff J.M., Akl E.A., Brennan S.E. (2021). The PRISMA 2020 statement: An updated guideline for reporting systematic reviews. BMJ.

[29] Adebisi Y.A., Alaran A.J., Bolarinwa O.A., Akande-Sholabi W., Lucero-Prisno D.E. (2021). When it is available, will we take it? Social media users’ perception of hypothetical COVID-19 vaccine in Nigeria. Pan Afr. Med. J..

[30] Ahmed M.A.M., Colebunders R., Gele A.A., Farah A.A., Osman S., Guled I.A., Abdullahi A.A.M., Hussein A.M., Ali A.M., Siewe Fodjo J.N. (2021). COVID-19 Vaccine Acceptability and Adherence to Preventive Measures in Somalia: Results of an Online Survey. Vaccines.

[31] Bongomin F., Olum R., Andia-Biraro I., Nakwagala F.N., Hassan K.H., Nassozi D.R., Kaddumukasa M., Byakika-Kibwika P., Kiguli S., Kirenga B.J. (2021). COVID-19 vaccine acceptance among high-risk populations in Uganda. Ther. Adv. Infect. Dis..

[32] Bono S.A., Faria de Moura Villela E., Siau C.S., Chen W.S., Pengpid S., Hasan M.T., Sessou P., Ditekemena J.D., Amodan B.O., Hosseinipour M.C. (2021). Factors Affecting COVID-19 Vaccine Acceptance: An International Survey among Low- and Middle-Income Countries. Vaccines.

[33] Carcelen A.C., Prosperi C., Mutembo S., Chongwe G., Mwansa F.D., Ndubani P., Simulundu E., Chilumba I., Musukwa G., Thuma P. (2022). COVID-19 vaccine hesitancy in Zambia: A glimpse at the possible challenges ahead for COVID-19 vaccination rollout in sub-Saharan Africa. Hum. Vaccin. Immunother..

[34] Carpio C.E., Sarasty O., Hudson D., Macharia A., Shibia M. (2021). The demand for a COVID-19 vaccine in Kenya. Hum. Vaccin. Immunother..

[35] Echoru I., Ajambo P.D., Keirania E., Bukenya E.E.M. (2021). Sociodemographic factors associated with acceptance of COVID-19 vaccine and clinical trials in Uganda: A cross-sectional study in western Uganda. BMC Public Health.

[36] Elgendy M.O., Abdelrahim M.E.A. (2021). Public awareness about coronavirus vaccine, vaccine acceptance, and hesitancy. J. Med. Virol..

[37] El-Sokkary R.H., El Seifi O.S., Hassan H.M., Mortada E.M., Hashem M.K., Gadelrab M.R.M.A., Tash R.M.E. (2021). Predictors of COVID-19 vaccine hesitancy among Egyptian healthcare workers: A cross-sectional study. BMC Infect. Dis..

[38] Hammam N., Tharwat S., Shereef R.R.E., Elsaman A.M., Khalil N.M., Fathi H.M., Salem M.N., El-Saadany H.M., Samy N., El-Bahnasawy A.S. (2021). Rheumatology university faculty opinion on coronavirus disease-19 (COVID-19) vaccines: The vaXurvey study from Egypt. Rheumatol. Int..

[39] Huynh G., Van Nguyen T., Nguyen D.D., Lam Q.M., Pham T.N., Nguyen H.T.N. (2021). Knowledge About COVID-19, Beliefs and Vaccination Acceptance Against COVID-19 Among High-Risk People in Ho Chi Minh City, Vietnam. Infect. Drug Resist..

[40] Jain J., Saurabh S., Kumar P., Verma M.K., Goel A.D., Gupta M.K., Bhardwaj P., Raghav P.R. (2021). COVID-19 vaccine hesitancy among medical students in India. Epidemiol. Infect..

[41] Kanyike A.M., Olum R., Kajjimu J., Ojilong D., Akech G.M., Nassozi D.R., Agira D., Wamala N.K., Asiimwe A., Matovu D. (2021). Acceptance of the coronavirus disease-2019 vaccine among medical students in Uganda. Trop. Med. Health.

[42] Kaur A., Kaur G., Kashyap A., Singh G., Singh Sandhu H., Khilji I., Singh Gambhir R. (2021). Attitude and acceptance of COVID-19 vaccine amongst medical and dental fraternity—A questionnaire survey. Rocz. Panstw. Zakl. Hig..

[43] Kitonsa J., Kamacooko O., Bahemuka U.M., Kibengo F., Kakande A., Wajja A., Basajja V., Lumala A., Ssemwanga E., Asaba R. (2021). Willingness to participate in COVID-19 vaccine trials; a survey among a population of healthcare workers in Uganda. PLoS ONE.

[44] Kumari A., Ranjan P., Chopra S., Kaur D., Kaur T., Upadhyay A.D., Isaac J.A., Kasiraj R., Prakash B., Kumar P. (2021). Knowledge, barriers and facilitators regarding COVID-19 vaccine and vaccination programme among the general population: A cross-sectional survey from one thousand two hundred and forty-nine participants. Diabetes Metab. Syndr..

[45] Lamptey E., Serwaa D., Appiah A.B. (2021). A nationwide survey of the potential acceptance and determinants of COVID-19 vaccines in Ghana. Clin. Exp. Vaccine Res..

[46] Lazarus J.V., Ratzan S.C., Palayew A., Gostin L.O., Larson H.J., Rabin K., Kimball S., El-Mohandes A. (2021). A global survey of potential acceptance of a COVID-19 vaccine. Nat. Med..

[47] Lazarus J.V., Wyka K., Rauh L., Rabin K., Ratzan S., Gostin L.O., Larson H.J., El-Mohandes A. (2020). Hesitant or Not? The Association of Age, Gender, and Education with Potential Acceptance of a COVID-19 Vaccine: A Country-level Analysis. J. Health Commun..

[48] Mohamad O., Zamlout A., AlKhoury N., Mazloum A.A., Alsalkini M., Shaaban R. (2021). Factors associated with the intention of Syrian adult population to accept COVID19 vaccination: A cross-sectional study. BMC Public Health.

[49] Panda D.S., Giri R.K., Nagarajappa A.K., Basha S. (2021). COVID-19 vaccine, acceptance, and concern of safety from public perspective in the state of Odisha, India. Hum. Vaccin. Immunother..

[50] Parvej M.I., Sultana S., Tabassum M., Mannan S.E., Ahmed F. (2021). Determinants of COVID-19 vaccine acceptance and encountered side-effects among the vaccinated in Bangladesh. Asian Pac. J. Trop. Med..

[51] Paudel S., Palaian S., Shankar P.R., Subedi N. (2021). Risk Perception and Hesitancy Toward COVID-19 Vaccination Among Healthcare Workers and Staff at a Medical College in Nepal. Risk Manag. Healthc. Policy.

[52] Qunaibi E.A., Helmy M., Basheti I., Sultan I. (2021). A high rate of COVID-19 vaccine hesitancy in a large-scale survey on Arabs. Elife.

[53] Ramesh Masthi N.R., Sowmyashree U. (2021). Awareness of COVID 19 vaccine in a rural area near Bangalore, Karnataka. Natl. J. Community Med..

[54] Saied S.M., Saied E.M., Kabbash I.A., Abdo S.A.E.-F. (2021). Vaccine hesitancy: Beliefs and barriers associated with COVID-19 vaccination among Egyptian medical students. J. Med. Virol..

[55] Skjefte M., Ngirbabul M., Akeju O., Escudero D., Hernandez-Diaz S., Wyszynski D.F., Wu J.W. (2021). COVID-19 vaccine acceptance among pregnant women and mothers of young children: Results of a survey in 16 countries. Eur. J. Epidemiol..

[56] Joanna Briggs Institute (2017). Checklist for Analytical Cross Sectional Studies, Critical Appraisal Tools. Joanna Briggs Inst..

[57] Al-Amer R., Maneze D., Everett B., Montayre J., Villarosa A.R., Dwekat E., Salamonson Y. (2021). COVID-19 vaccination intention in the first year of the pandemic: A systematic review. J. Clin. Nurs..

[58] Villarosa A.R., Maneze D., Ramjan L.M., Srinivas R., Camilleri M., George A. (2019). The effectiveness of guideline implementation strategies in the dental setting: A systematic review. Implement. Sci..

[59] Higgins J.P.T., Thompson S.G. (2002). Quantifying heterogeneity in a meta-analysis. Stat. Med..

[60] Patwary M.M., Hossain M.R., Shuvo F.K., Ashraf S., Sultana R., Alam M.A. (2021). Protecting Sanitation Workers in Low-Middle Income Countries Amid COVID-19. Ann. Work Expo. Health.

[61] Anwar S., Nasrullah M., Hosen M.J. (2020). COVID-19 and Bangladesh: Challenges and How to Address Them. Front. Public Health.

[62] Wouters O.J., Shadlen K.C., Salcher-Konrad M., Pollard A.J., Larson H.J., Teerawattananon Y., Jit M. (2021). Challenges in ensuring global access to COVID-19 vaccines: Production, affordability, allocation, and deployment. Lancet.

[63] Maeda J.M., Nkengasong J.N. (2021). The puzzle of the COVID-19 pandemic in Africa. Science.

[64] Christensen D., Dube O., Haushofer J., Siddiqi B., Voors M. (2021). Building resilient health systems: Experimental evidence from sierra leone and the 2014 ebola outbreak. Q. J. Econ..

[65] Barello S., Nania T., Dellafiore F., Graffigna G., Caruso R. (2020). “Vaccine hesitancy” among university students in Italy during the COVID-19 pandemic. Eur. J. Epidemiol..

[66] The Business Standard India’s Omicron Surge Explained: Reproduction Number up, Doubling Time down | Business Standard News. https://www.business-standard.com/article/current-affairs/india-s-omicron-surge-explained-reproduction-number-up-doubling-time-down-122010900082_1.html.

[67] Kabamba Nzaji M., Kabamba Ngombe L., Ngoie Mwamba G., Banza Ndala D.B., Mbidi Miema J., Luhata Lungoyo C., Lora Mwimba B., Cikomola Mwana Bene A., Mukamba Musenga E. (2020). Acceptability of Vaccination Against COVID-19 Among Healthcare Workers in the Democratic Republic of the Congo. Pragmatic Obs. Res..

[68] Jegede A.S. (2007). What led to the Nigerian boycott of the polio vaccination campaign?. PLoS Med..

[69] Dzinamarira T., Nachipo B., Phiri B., Musuka G. (2021). COVID-19 vaccine roll-out in South Africa and Zimbabwe: Urgent need to address community preparedness, fears and hesitancy. Vaccines.

[70] Goldman R.D., Yan T.D., Seiler M., Parra Cotanda C., Brown J.C., Klein E.J., Hoeffe J., Gelernter R., Hall J.E., Davis A.L. (2020). Caregiver willingness to vaccinate their children against COVID-19: Cross sectional survey. Vaccine.

[71] Jiménez-García R., Hernández-Barrera V., de Andres A.L., Jimenez-Trujillo I., Esteban-Hernández J., Carrasco-Garrido P. (2010). Gender influence in influenza vaccine uptake in Spain: Time trends analysis (1995–2006). Vaccine.

[72] Sallam M., Dababseh D., Yaseen A., Al-Haidar A., Taim D., Eid H., Ababneh N.A., Bakri F.G., Mahafzah A. (2020). COVID-19 misinformation: Mere harmless delusions or much more? A knowledge and attitude cross-sectional study among the general public residing in Jordan. PLoS ONE.

[73] Nindrea R.D., Usman E., Katar Y., Sari N.P. (2021). Acceptance of COVID-19 vaccination and correlated variables among global populations: A systematic review and meta-analysis. Clin. Epidemiol. Glob. Health.

[74] Rajamoorthy Y., Radam A., Taib N.M., Rahim K.A., Wagner A.L., Mudatsir M., Munusamy S., Harapan H. (2018). The relationship between perceptions and self-paid hepatitis B vaccination: A structural equation modeling approach. PLoS ONE.

[75] Sundaram N., Purohit V., Schaetti C., Kudale A., Joseph S., Weiss M.G. (2015). Community awareness, use and preference for pandemic influenza vaccines in pune, India. Hum. Vaccines Immunother..

[76] Wong L.P., Alias H., Wong P.-F., Lee H.Y., AbuBakar S. (2020). The use of the health belief model to assess predictors of intent to receive the COVID-19 vaccine and willingness to pay. Hum. Vaccin. Immunother..

[77] Wu D.C., Jha P., Lam T., Brown P., Gelband H., Nagelkerke N., Birnboim H.C., Reid A. (2020). Predictors of self-reported symptoms and testing for COVID-19 in Canada using a nationally representative survey. PLoS ONE.

[78] Johnson N.F., Velásquez N., Restrepo N.J., Leahy R., Gabriel N., El Oud S., Zheng M., Manrique P., Wuchty S., Lupu Y. (2020). The online competition between pro- and anti-vaccination views. Nature.

[79] Daly M., Robinson E. (2021). Willingness to Vaccinate against COVID-19 in the U.S.: Representative Longitudinal Evidence from April to October 2020. Am. J. Prev. Med..

[80] Wright K.B. (2005). Researching internet-based populations: Advantages and disadvantages of online survey research, online questionnaire authoring software packages, and web survey services. J. Comput. Commun..

