# Supplementary material for: COVID-19 Vaccine Acceptance among Low- and Lower-Middle-Income Countries: A Rapid Systematic Review and Meta-Analysis
Source: Vaccines (Basel). 2022 Mar 11;10(3):427. doi: 10.3390/vaccines10030427 (PMC8950670; doi:10.3390/vaccines10030427)
Supplement: Supplementary file 1 [file vaccines-10-00427-s001.zip › vaccines-1610997-supplementary.pdf]

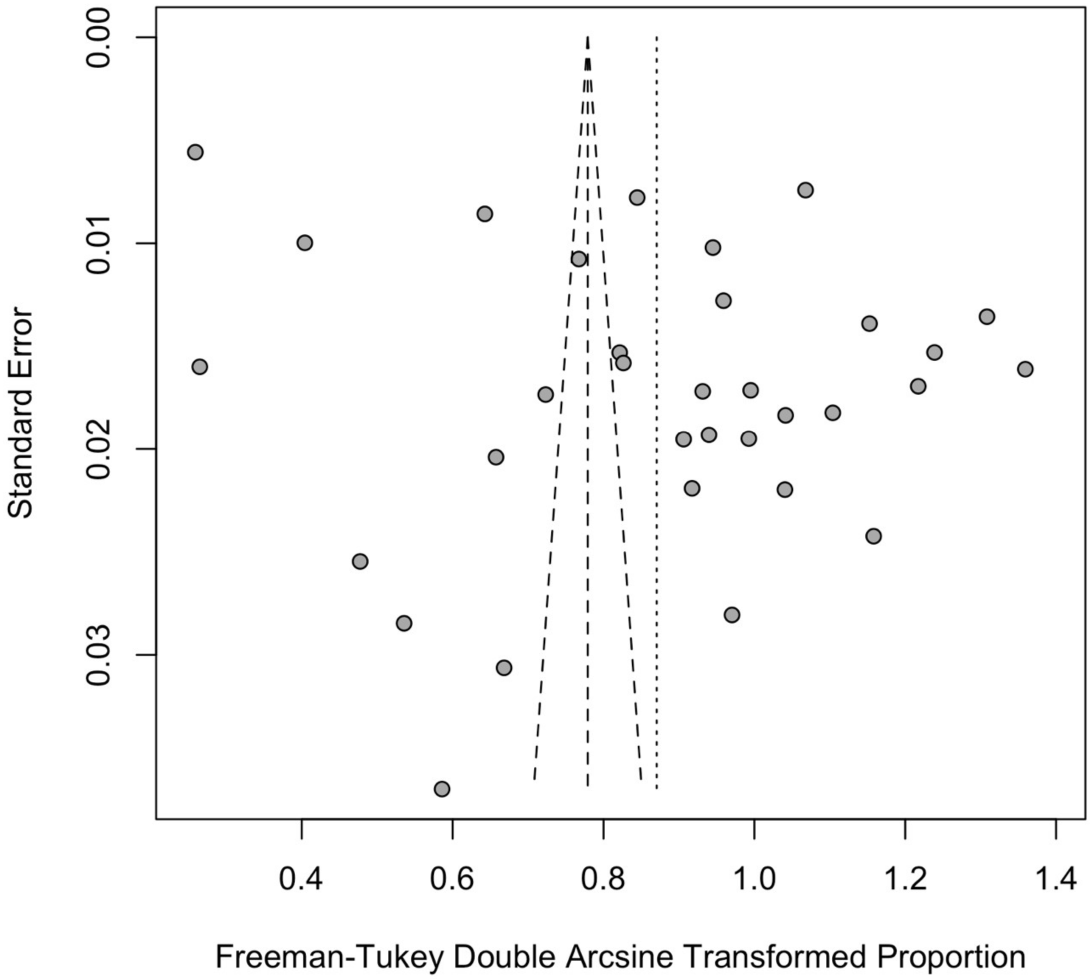

Figure S1. Funnel plot of vaccine acceptance.

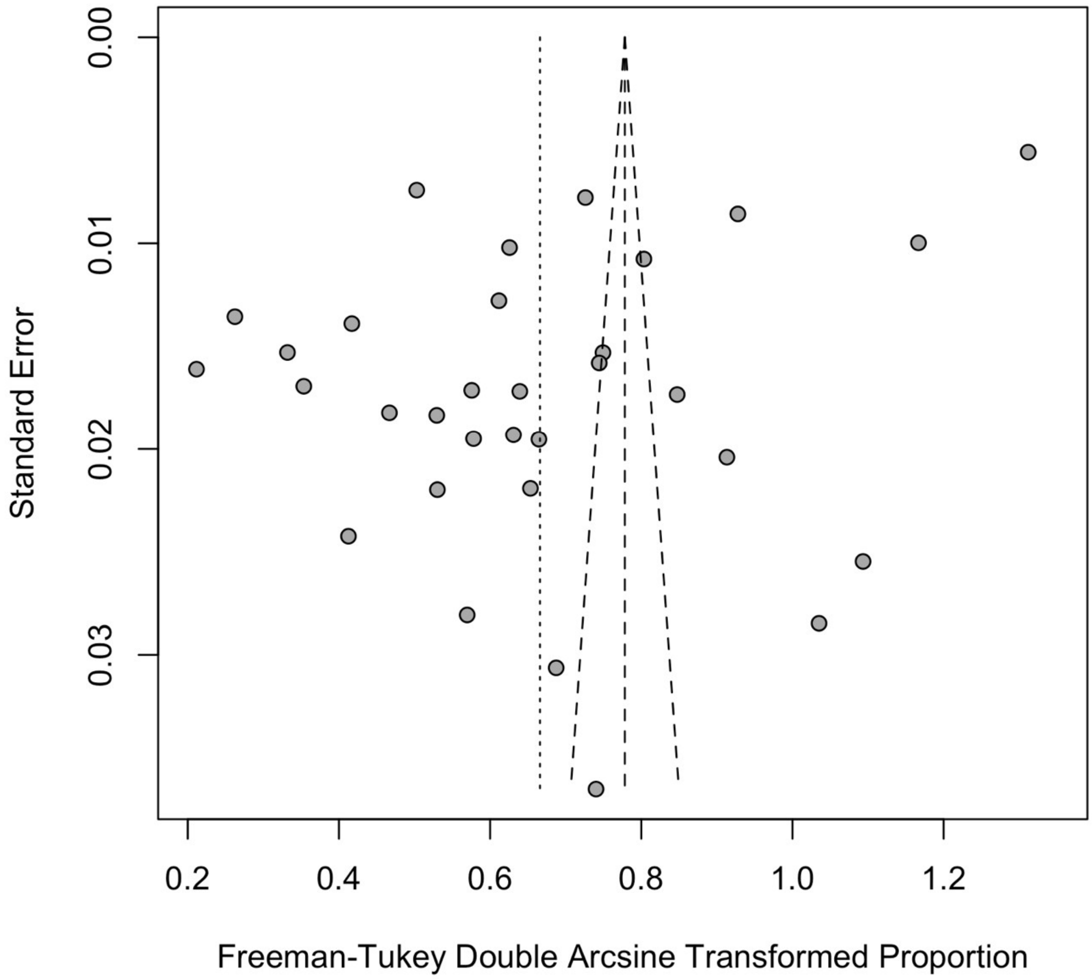

Figure S2. Funnel plot of vaccine hesitance.

Table S1. Risk of bias for included studies in the systematic review.

SL	Author Reference	1. Were the Criteria for Inclusion in the Sample Clearly Defined?	2. Were the Study Subjects and the Setting Described in Detail?	3. Was the Exposure Measured in a Valid and Reliable Way?	4. Were Objective, Standard Criteria Used for Measurement of the Condition?	5. Were Confounding Factors Identified?	6. Were Strategies to Deal with Confounding Factors Stated?	7. Were the Outcomes Measured in a Valid and Reliable Way?	8. Was Appropriate Statistical Analysis Used?	Total (%)	Quality Level (High: ≥50%; Low: <50%)
1	Adebisi et al. [29]	1	1	1	1	0	NC	0	1	62.5	High
2	Ahmed et al. [30]	1	1	1	1	0	NC	0	1	62.5	High
3	Ahmed et al. [20]	1	1	1	1	0	NC	0	1	62.5	High
4	Akiful Haque et al. [17]	1	1	1	1	0	0	1	1	75	High
5	Alam et al. [18]	1	1	1	1	1	1	0	1	87.5	High
6	Arshad et al. [19]	1	1	1	0	0	0	0	1	50	High
7	Bongomin et al. [31]	1	1	1	0	0	0	0	1	50	High
8	Bono et al. [32]	1	1	1	0	0	0	1	1	62.5	High
9	Bono et al. [32]	1	1	1	1	0	0	1	1	75	High

10	Carcelen et al. [33]	1	1	1	1	0	0	1	1	75	High
11	Carpio et al. [34]	1	1	1	1	0	0	1	1	75	High
12	Dinga et al. [21]	1	1	1	0	0	0	1	1	62.5	High
13	Echoru et al. [35]	1	1	1	1	0	0	1	0	62.5	High
14	Elgendy and Abdelrahim [36]	1	1	1	1	0	0	1	1	75	High
15	El-Sokkary et al. [37]	1	1	1	1	1	1	1	1	100	High
16	Fares et al. [22]	1	1	1	1	0	0	0	1	62.5	High
17	Hammam et al. [38]	1	1	1	1	0	0	1	1	75	High
18	Harapan et al. [8]	1	1	1	1	0	0	1	1	75	High
19	Huynh et al. [39]	1	1	1	0	0	0	0	1	50	High
20	Jain et al. [40]	1	1	1	0	0	0	0	1	50	High
21	Kanyike et al. [41]	1	1	1	1	0	0	1	1	75	High
22	Kaur et al. [42]	1	1	1	1	0	0	1	1	75	High
23	Kitonsa et al. [43]	1	1	1	1	0	0	0	1	62.5	High

24	Kumari et al. [44]	1	1	1	1	0	0	1	1	75	High
25	Lamptey et al. [45]	1	1	1	1	0	0	1	1	75	High
26	Lazarus et al. [46]	1	1	1	1	0	0	1	1	75	High
27	Lazarus et al. [47]	1	1	1	1	0	0	0	1	62.5	High
28	Mohamad et al. [48]	1	1	1	1	0	0	0	1	62.5	High
29	Panda et al. [49]	1	1	1	1	0	0	1	0	62.5	High
30	Parvej et al. [50]	1	1	1	0	1	1	1	1	87.5	High
31	Paudel et al. [51]	1	1	1	0	0	0	0	1	50	High
32	Qunaibi et al. [52]	1	1	0	1	0	0	0	1	50	High
33	Ramesh Masthi and Sowmyashree [53]	1	1	1	0	0	0	0	1	50	High
34	Saied et al. [54]	1	1	0	1	0	0	1	1	62.5	High
35	Skjefte et al. [55]	1	1	1	1	0	0	0	1	62.5	High
36	Solis Arce et al. [15]	1	1	1	1	0	0	0	1	62.5	High

Yes (1); No (0); Not clear (NC).

Table S2. Tests for publication bias

Outcome	No. of Studies	Egger's *p*-Value	Interpretation
Acceptance	33	0.02	Eggers' test indicates the presence of funnel plot asymmetry.
Hesitancy	32	0.007	Eggers' test indicates the presence of funnel plot asymmetry.
